# 4-(4-Hydroxybenzoyl)phenol mono­hydrate

**DOI:** 10.1107/S1600536810041425

**Published:** 2010-10-20

**Authors:** He-Ping Li, Yun-Xia Yang, Seik Weng Ng

**Affiliations:** a Key Laboratory of Polymer Materials of Gansu Province Ministry of Education, College of Chemistry and Chemical Engineering, Northwest Normal University, Lanzhou 730070, Gansu, People’s Republic of China; b Department of Chemistry, University of Malaya, 50603 Kuala Lumpur, Malaysia

## Abstract

The aromatic rings of the title compound, C_13_H_10_O_3_·H_2_O, are aligned at dihedral angles of 20.6 (1) and 40.8 (1)° with respect to the triangular C_ar­yl_–C(=O)–C_ar­yl_ fragment. The hy­droxy groups are each hydrogen-bond donors to separate water mol­ecules, the water mol­ecule itself being hydrogen-bonded to one hy­droxy group and one carbonyl group. The water mol­ecule exists in an unusual four-coordinate environment in the resulting layer structure.

## Related literature

For the crystal structure of anhydrous 4,4′-dihy­droxy­benzophenone, see: Ferguson & Glidewell (1996 ▶).
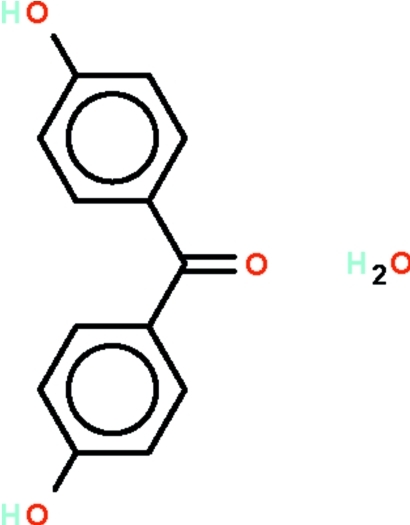


## Experimental

### 

#### Crystal data

C_13_H_10_O_3_·H_2_O
*M* *_r_* = 232.23
Monoclinic, 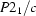

*a* = 4.9398 (1) Å
*b* = 9.8273 (2) Å
*c* = 23.1446 (4) Å
β = 94.520 (1)°
*V* = 1120.06 (4) Å^3^
*Z* = 4
Mo *K*α radiation
μ = 0.10 mm^−1^
*T* = 293 K
0.45 × 0.30 × 0.05 mm

#### Data collection

Bruker SMART APEX diffractometer
8356 measured reflections
2572 independent reflections
2016 reflections with *I* > 2σ(*I*)
*R*_int_ = 0.017

#### Refinement

*R*[*F* ^2^ > 2σ(*F* ^2^)] = 0.040
*wR*(*F* ^2^) = 0.123
*S* = 1.05
2572 reflections
170 parameters
4 restraints
H atoms treated by a mixture of independent and constrained refinement
Δρ_max_ = 0.22 e Å^−3^
Δρ_min_ = −0.17 e Å^−3^

### 

Data collection: *APEX2* (Bruker, 2007 ▶); cell refinement: *SAINT* (Bruker, 2007 ▶); data reduction: *SAINT*; program(s) used to solve structure: *SHELXS97* (Sheldrick, 2008 ▶); program(s) used to refine structure: *SHELXL97* (Sheldrick, 2008 ▶); molecular graphics: *X-SEED* (Barbour, 2001 ▶); software used to prepare material for publication: *publCIF* (Westrip, 2010 ▶).

## Supplementary Material

Crystal structure: contains datablocks global, I. DOI: 10.1107/S1600536810041425/zs2075sup1.cif

Structure factors: contains datablocks I. DOI: 10.1107/S1600536810041425/zs2075Isup2.hkl

Additional supplementary materials:  crystallographic information; 3D view; checkCIF report

## Figures and Tables

**Table 1:** Hydrogen-bond geometry (Å, °)

*D*—H⋯*A*	*D*—H	H⋯*A*	*D*⋯*A*	*D*—H⋯*A*
O1—H1⋯O1w^i^	0.85 (1)	1.95 (1)	2.774 (2)	164 (2)
O3—H3⋯O1w^ii^	0.85 (1)	1.95 (1)	2.773 (2)	164 (2)
O1w—H11⋯O2	0.84 (1)	1.93 (1)	2.762 (2)	168 (2)
O1w—H12⋯O1^iii^	0.84 (1)	2.18 (2)	2.898 (2)	143 (2)

Symmetry codes: (i) 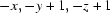; (ii) 
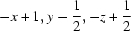
; (iii) 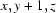.

**Fig. 1.**
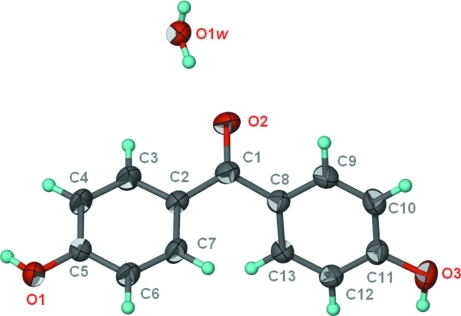
Thermal ellipsoid plot (Barbour, 2001) of C13H10O3.H2O at the 50% probability level.

**Fig. 2.**
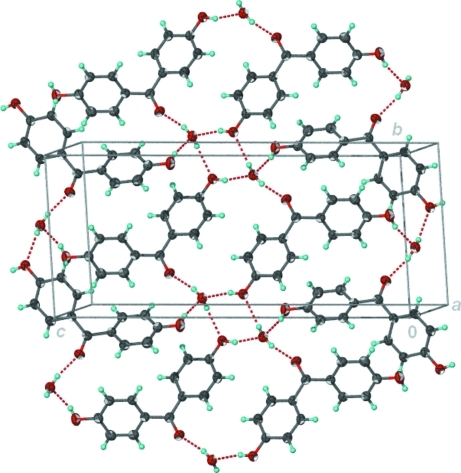
The layer structure of the title compound with hydrogen-bonding interactions shown as dashed lines.

## supplementary crystallographic information

### Comment

4,4'-Dihydroxbenzophenone exists as a *O*–*H*···O_hydroxy_ hydrogen-bonded chains that are linked by *O*–*H*···O_carbonyl_ hydrogen bonds into sheets. The first set of hydrogen bonds [2.785 (4), 2.791 (4) Å] is longer than the second set [2.624 (4), 2.627 (4) Å] (Ferguson & Glidewell, 1996). The monohydrated title compound C_13_H_10_O_3_^.^H_2_O (Scheme I, Fig. 1) also adopts a hydrogen-bonded sheet motif. The aromatic rings are aligned at 20.6 (1) and 40.8 (1) ° with respect to the triangular-shaped C_aryl_–C(═*O*)–C_aryl_ fragment. The hydroxy groups are each hydrogen-bond donors to separate water molecules which also act as hydrogen-bond donors to an hydroxy group and a carbonyl group (Table 1). There are no hydroxy···carbonyl interactions, unlike those found in the anhydrous compound. The water molecule exists in an unusual four-coordinate environment in the resulting two-dimensional layer structure (Fig. 2).

### Experimental

Anhydrous 4,4'-dihydroxybenzophenone (0.25 mmol, 0.054 g) and boric acid (0.50 mmol, 0.031 g) were dissolved in a water-ethanol mixture (50 ml/100 ml *v*/*v*). Trimethylamine (33% aqueous solution) was added until the solution registered a neutral pH. The mixture was then set aside for a few days after which yellow crystal blocks of the title compound were isolated.

### Refinement

Carbon-bound H-atoms were placed in calculated positions (C–H = 0.93 Å) and were included in the refinement in the riding model approximation, with *U*_iso_(H) set to 1.2*U*_eq_(C). The hydroxy and water H-atoms were located in a difference Fourier map, and were included in the refinement with a distance restraint of O–H = 0.84±0.01 Å and with their isotropic displacement parameters refined.

### Crystal data

**Table:**

C_13_H_10_O_3_·H_2_O	*F*(000) = 488
*M**_r_* = 232.23	*D*_x_ = 1.377 Mg m^−^^3^
Monoclinic, *P*2_1_/*c*	Mo *K*α radiation, λ = 0.71073 Å
Hall symbol: -P 2ybc	Cell parameters from 3494 reflections
*a* = 4.9398 (1) Å	θ = 2.7–27.2°
*b* = 9.8273 (2) Å	µ = 0.10 mm^−^^1^
*c* = 23.1446 (4) Å	*T* = 293 K
β = 94.520 (1)°	Block, yellow
*V* = 1120.06 (4) Å^3^	0.45 × 0.30 × 0.05 mm
*Z* = 4	

### Data collection

**Table:**

Bruker SMART APEX diffractometer	2016 reflections with *I* > 2σ(*I*)
Radiation source: fine-focus sealed tube	*R*_int_ = 0.017
graphite	θ_max_ = 27.5°, θ_min_ = 1.8°
ω scans	*h* = −6→6
8356 measured reflections	*k* = −11→12
2572 independent reflections	*l* = −29→29

### Refinement

**Table:**

Refinement on *F*^2^	Primary atom site location: structure-invariant direct methods
Least-squares matrix: full	Secondary atom site location: difference Fourier map
*R*[*F*^2^ > 2σ(*F*^2^)] = 0.040	Hydrogen site location: inferred from neighbouring sites
*wR*(*F*^2^) = 0.123	H atoms treated by a mixture of independent and constrained refinement
*S* = 1.05	*w* = 1/[σ^2^(*F*_o_^2^) + (0.0574*P*)^2^ + 0.2426*P*] where *P* = (*F*_o_^2^ + 2*F*_c_^2^)/3
2572 reflections	(Δ/σ)_max_ = 0.001
170 parameters	Δρ_max_ = 0.22 e Å^−^^3^
4 restraints	Δρ_min_ = −0.17 e Å^−^^3^

### Special details

**Table:**

Geometry. All e.s.d.'s (except the e.s.d. in the dihedral angle between two l.s. planes) are estimated using the full covariance matrix. The cell e.s.d.'s are taken into account individually in the estimation of e.s.d.'s in distances, angles and torsion angles; correlations between e.s.d.'s in cell parameters are only used when they are defined by crystal symmetry. An approximate (isotropic) treatment of cell e.s.d.'s is used for estimating e.s.d.'s involving l.s. planes.

### Fractional atomic coordinates and isotropic or equivalent isotropic displacement parameters (Å^2^)

**Table:**

	*x*	*y*	*z*	*U*_iso_*/*U*_eq_	
O1	0.2714 (2)	0.12226 (11)	0.49586 (5)	0.0570 (3)	
O2	0.5003 (3)	0.68859 (12)	0.38317 (5)	0.0671 (4)	
O3	1.0143 (3)	0.54977 (14)	0.15066 (5)	0.0634 (3)	
O1W	0.1638 (3)	0.86726 (12)	0.43550 (5)	0.0554 (3)	
C1	0.5324 (3)	0.57501 (15)	0.36223 (6)	0.0465 (3)	
C2	0.4542 (3)	0.45204 (15)	0.39381 (6)	0.0442 (3)	
C3	0.2632 (4)	0.46347 (17)	0.43426 (8)	0.0676 (5)	
H3A	0.1772	0.5466	0.4387	0.081*	
C4	0.1979 (4)	0.35531 (17)	0.46788 (8)	0.0668 (5)	
H4	0.0674	0.3653	0.4944	0.080*	
C5	0.3249 (3)	0.23209 (15)	0.46244 (6)	0.0454 (3)	
C6	0.5141 (3)	0.21717 (16)	0.42219 (7)	0.0536 (4)	
H6	0.5993	0.1338	0.4180	0.064*	
C7	0.5768 (3)	0.32592 (16)	0.38821 (6)	0.0508 (4)	
H7	0.7037	0.3148	0.3610	0.061*	
C8	0.6547 (3)	0.56332 (14)	0.30623 (6)	0.0426 (3)	
C9	0.8488 (3)	0.65824 (16)	0.29241 (7)	0.0509 (4)	
H9	0.8994	0.7271	0.3186	0.061*	
C10	0.9667 (3)	0.65163 (17)	0.24064 (7)	0.0552 (4)	
H10	1.1004	0.7139	0.2326	0.066*	
C11	0.8866 (3)	0.55235 (15)	0.20044 (6)	0.0459 (3)	
C12	0.6853 (3)	0.46052 (15)	0.21229 (6)	0.0457 (3)	
H12A	0.6253	0.3962	0.1847	0.055*	
C13	0.5745 (3)	0.46505 (14)	0.26519 (6)	0.0451 (3)	
H13	0.4440	0.4013	0.2735	0.054*	
H1	0.147 (4)	0.141 (2)	0.5179 (8)	0.091 (7)*	
H3	0.946 (5)	0.4859 (19)	0.1296 (9)	0.103 (9)*	
H11	0.260 (4)	0.8047 (19)	0.4233 (10)	0.101 (8)*	
H12	0.262 (5)	0.921 (2)	0.4565 (10)	0.111 (9)*	

### Atomic displacement parameters (Å^2^)

**Table:**

	*U*^11^	*U*^22^	*U*^33^	*U*^12^	*U*^13^	*U*^23^
O1	0.0723 (7)	0.0453 (6)	0.0572 (7)	−0.0005 (5)	0.0286 (6)	0.0009 (5)
O2	0.1019 (10)	0.0421 (6)	0.0609 (7)	0.0052 (6)	0.0298 (7)	−0.0063 (5)
O3	0.0714 (8)	0.0729 (8)	0.0487 (6)	−0.0131 (6)	0.0223 (6)	−0.0028 (6)
O1W	0.0679 (7)	0.0496 (7)	0.0508 (6)	−0.0027 (6)	0.0174 (6)	−0.0040 (5)
C1	0.0537 (8)	0.0424 (8)	0.0440 (7)	0.0039 (6)	0.0080 (6)	−0.0038 (6)
C2	0.0493 (8)	0.0432 (8)	0.0413 (7)	0.0013 (6)	0.0103 (6)	−0.0042 (6)
C3	0.0859 (12)	0.0445 (9)	0.0784 (12)	0.0133 (8)	0.0452 (10)	−0.0004 (8)
C4	0.0808 (12)	0.0522 (9)	0.0741 (11)	0.0072 (8)	0.0478 (10)	−0.0008 (8)
C5	0.0519 (8)	0.0429 (8)	0.0429 (7)	−0.0032 (6)	0.0118 (6)	−0.0021 (6)
C6	0.0650 (9)	0.0450 (8)	0.0539 (9)	0.0108 (7)	0.0234 (7)	0.0010 (7)
C7	0.0582 (9)	0.0501 (8)	0.0470 (8)	0.0083 (7)	0.0223 (7)	0.0007 (6)
C8	0.0485 (7)	0.0388 (7)	0.0411 (7)	0.0031 (6)	0.0074 (6)	0.0012 (6)
C9	0.0605 (9)	0.0439 (8)	0.0486 (8)	−0.0085 (7)	0.0064 (7)	−0.0056 (6)
C10	0.0595 (9)	0.0514 (9)	0.0559 (9)	−0.0154 (7)	0.0123 (7)	−0.0002 (7)
C11	0.0505 (8)	0.0468 (8)	0.0413 (7)	0.0020 (6)	0.0089 (6)	0.0052 (6)
C12	0.0539 (8)	0.0426 (8)	0.0408 (7)	−0.0022 (6)	0.0045 (6)	−0.0030 (6)
C13	0.0497 (8)	0.0407 (7)	0.0458 (7)	−0.0041 (6)	0.0088 (6)	−0.0001 (6)

### Geometric parameters (Å, °)

**Table:**

O1—C5	1.3659 (17)	C5—C6	1.3784 (19)
O1—H1	0.847 (10)	C6—C7	1.377 (2)
O2—C1	1.2322 (18)	C6—H6	0.9300
O3—C11	1.3566 (17)	C7—H7	0.9300
O3—H3	0.847 (10)	C8—C13	1.390 (2)
O1W—H11	0.841 (10)	C8—C9	1.393 (2)
O1W—H12	0.844 (10)	C9—C10	1.375 (2)
C1—C8	1.4771 (19)	C9—H9	0.9300
C1—C2	1.479 (2)	C10—C11	1.384 (2)
C2—C3	1.385 (2)	C10—H10	0.9300
C2—C7	1.390 (2)	C11—C12	1.386 (2)
C3—C4	1.371 (2)	C12—C13	1.3810 (19)
C3—H3A	0.9300	C12—H12A	0.9300
C4—C5	1.374 (2)	C13—H13	0.9300
C4—H4	0.9300		
			
C5—O1—H1	110.4 (16)	C6—C7—C2	121.32 (13)
C11—O3—H3	108.2 (17)	C6—C7—H7	119.3
H11—O1W—H12	110 (2)	C2—C7—H7	119.3
O2—C1—C8	119.34 (13)	C13—C8—C9	118.22 (13)
O2—C1—C2	119.92 (13)	C13—C8—C1	122.62 (13)
C8—C1—C2	120.73 (12)	C9—C8—C1	119.08 (13)
C3—C2—C7	117.41 (14)	C10—C9—C8	121.01 (14)
C3—C2—C1	119.09 (13)	C10—C9—H9	119.5
C7—C2—C1	123.34 (12)	C8—C9—H9	119.5
C4—C3—C2	121.61 (15)	C9—C10—C11	120.05 (14)
C4—C3—H3A	119.2	C9—C10—H10	120.0
C2—C3—H3A	119.2	C11—C10—H10	120.0
C3—C4—C5	120.11 (14)	O3—C11—C10	117.16 (13)
C3—C4—H4	119.9	O3—C11—C12	122.99 (14)
C5—C4—H4	119.9	C10—C11—C12	119.84 (13)
O1—C5—C4	122.29 (12)	C13—C12—C11	119.68 (13)
O1—C5—C6	118.08 (13)	C13—C12—H12A	120.2
C4—C5—C6	119.64 (14)	C11—C12—H12A	120.2
C7—C6—C5	119.90 (14)	C12—C13—C8	121.10 (13)
C7—C6—H6	120.1	C12—C13—H13	119.5
C5—C6—H6	120.1	C8—C13—H13	119.5
			
O2—C1—C2—C3	22.9 (2)	O2—C1—C8—C13	−144.18 (16)
C8—C1—C2—C3	−158.45 (16)	C2—C1—C8—C13	37.2 (2)
O2—C1—C2—C7	−152.26 (16)	O2—C1—C8—C9	32.5 (2)
C8—C1—C2—C7	26.4 (2)	C2—C1—C8—C9	−146.10 (15)
C7—C2—C3—C4	0.4 (3)	C13—C8—C9—C10	−2.8 (2)
C1—C2—C3—C4	−175.06 (18)	C1—C8—C9—C10	−179.63 (15)
C2—C3—C4—C5	0.8 (3)	C8—C9—C10—C11	2.2 (3)
C3—C4—C5—O1	178.47 (18)	C9—C10—C11—O3	−178.83 (15)
C3—C4—C5—C6	−1.3 (3)	C9—C10—C11—C12	0.6 (2)
O1—C5—C6—C7	−179.09 (15)	O3—C11—C12—C13	176.64 (14)
C4—C5—C6—C7	0.7 (3)	C10—C11—C12—C13	−2.8 (2)
C5—C6—C7—C2	0.5 (3)	C11—C12—C13—C8	2.2 (2)
C3—C2—C7—C6	−1.0 (3)	C9—C8—C13—C12	0.6 (2)
C1—C2—C7—C6	174.23 (15)	C1—C8—C13—C12	177.31 (14)

### Hydrogen-bond geometry (Å, °)

**Table:**

*D*—H···*A*	*D*—H	H···*A*	*D*···*A*	*D*—H···*A*
O1—H1···O1w^i^	0.85 (1)	1.95 (1)	2.774 (2)	164 (2)
O3—H3···O1w^ii^	0.85 (1)	1.95 (1)	2.773 (2)	164 (2)
O1w—H11···O2	0.84 (1)	1.93 (1)	2.762 (2)	168 (2)
O1w—H12···O1^iii^	0.84 (1)	2.18 (2)	2.898 (2)	143 (2)

Symmetry codes: (i) −*x*, −*y*+1, −*z*+1; (ii) −*x*+1, *y*−1/2, −*z*+1/2; (iii) *x*, *y*+1, *z*.
